# 203. The Path Toward Treatment: Identifying Barriers to Hepatitis C Care Linkage After Positive Emergency Department Screening

**DOI:** 10.1093/ofid/ofaf695.075

**Published:** 2026-01-11

**Authors:** Emma Nedell, Matthew Reppucci, David Plourde, Christian Klaucke, Meelynn Wong, Brenda Figueroa, Idelisa Turcios, Alexandra Rock, Jennifer Edwards, Martin Reznek, Thomas C Greenough

**Affiliations:** UMass Chan Medical School, Worcester, MA; UMass Chan Medical School, Worcester, MA; UMass Chan Medical School, Worcester, MA; UMass Chan Medical School, Worcester, MA; UMass Chan Medical School, Worcester, MA; UMass Memorial Health, Worcester, Massachusetts; UMass Memorial Health, Worcester, Massachusetts; UMass Memorial Health, Worcester, Massachusetts; UMass Chan Medical School, Worcester, MA; UMass Chan Medical School, Worcester, MA; UMass Memorial Medical Center, Worcester, Massachusetts

## Abstract

**Background:**

Half of individuals with hepatitis C virus (HCV) infection in the United States are unaware of their status. Expanding screening, linkage to care (LTC), and access to curative antiviral treatment are essential to reduce HCV-related morbidity and mortality and prevent continued transmission. This study investigates the HCV care cascade of an emergency department (ED)-based HCV screening program at UMass Memorial Health (UMMH). Factors associated with LTC and the impact of the COVID-19 pandemic on the care cascade were examined.

**Figure 1.**
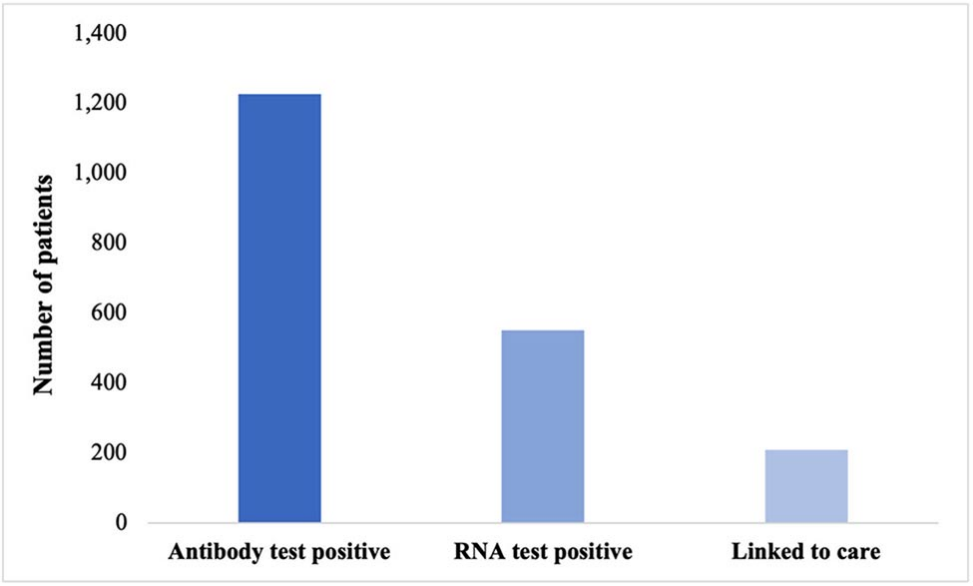
UMMH HCV care cascade, 10/2018-10/2022.

**Table 1.**
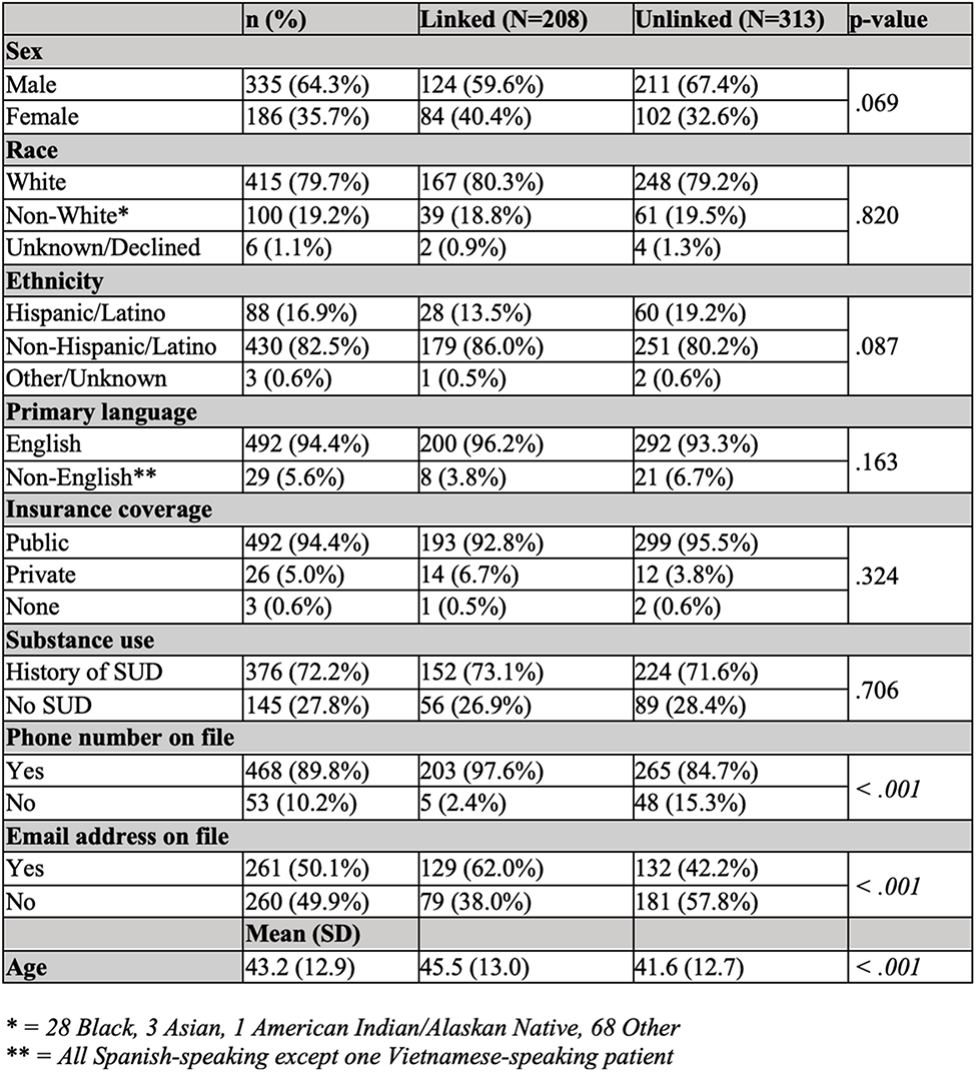
Sociodemographic characteristics of HCV RNA+ individuals (N=521) by linkage status.

**Methods:**

We performed a retrospective review of individuals with HCV antibody and RNA tests completed in five EDs from October 2018 to October 2022. Electronic health record data of patients with active HCV infection, indicated by positive RNA test, were abstracted. Chi-square tests were performed to identify differences in sociodemographic characteristics between linked (L) and unlinked (UL) patients as well as LTC rates before and during COVID-19.

**Results:**

Of 15,093 patients screened, 1,226 (8.1%) were HCV antibody positive. Of these, 550 (40.2%) were HCV RNA positive; 208 (39.9%) were linked to care and 313 (60.1%) were UL (Figure 1). A higher proportion of UL patients were male (67.4% UL vs. 59.6% L), Hispanic/Latino (19.2% UL vs. 13.5% L), and spoke a language other than English (6.7% UL vs. 3.8% L), though these trends were not statistically significant. On average, UL patients were younger (41.6 UL vs. 45.5 L; p < 0.001). Fewer UL patients had a phone number (84.7% UL vs. 97.6% L; p < 0.001) or email address (42.2% UL vs. 62.0% L; p < 0.001) recorded in their chart (Table 1). Furthermore, the COVID-19 pandemic impacted the HCV care cascade at UMMH. Fewer antibody (76.1 vs. 78.6) and RNA tests (6.9 vs. 8.1) were performed weekly after the pandemic began, and patients screened before the start of the pandemic were significantly more likely to be linked (46.6%) than those screened during (35.6%) (X2 (1, N=521) = 6.2, p = 0.013).

**Conclusion:**

Our findings highlight the need for LTC strategies that can be adapted to meet each patient where they are. Access to phone and internet services is crucial, particularly in circumstances where health systems are strained. Our analysis indicates that LTC strategies that consider age, gender, ethnicity, and language will likely be more effective.

**Disclosures:**

All Authors: No reported disclosures

